# Accelerated turnover of taste bud cells in mice deficient for the cyclin-dependent kinase inhibitor p27^Kip1^

**DOI:** 10.1186/1471-2202-12-34

**Published:** 2011-04-20

**Authors:** Theresa A Harrison, Lorraine B Smith Adams, Preston D Moore, Marla K Perna, Jarrod D Sword, Dennis M Defoe

**Affiliations:** 1 Department of Anatomy and Cell Biology, Quillen College of Medicine, East Tennessee State University, Johnson City, TN, USA; 2 Biomedical Science Graduate Program, Quillen College of Medicine, East Tennessee State University, Johnson City, TN, USA

## Abstract

**Background:**

Mammalian taste buds contain several specialized cell types that coordinately respond to tastants and communicate with sensory nerves. While it has long been appreciated that these cells undergo continual turnover, little is known concerning how adequate numbers of cells are generated and maintained. The cyclin-dependent kinase inhibitor p27^Kip1 ^has been shown to influence cell number in several developing tissues, by coordinating cell cycle exit during cell differentiation. Here, we investigated its involvement in the control of taste cell replacement by examining adult mice with targeted ablation of the *p27^Kip1 ^*gene.

**Results:**

Histological and morphometric analyses of fungiform and circumvallate taste buds reveal no structural differences between wild-type and *p27^Kip1^*-null mice. However, when examined in functional assays, mutants show substantial proliferative changes. In BrdU incorporation experiments, more S-phase-labeled precursors appear within circumvallate taste buds at 1 day post-injection, the earliest time point examined. After 1 week, twice as many labeled intragemmal cells are present, but numbers return to wild-type levels by 2 weeks. Mutant taste buds also contain more TUNEL-labeled cells and 50% more apoptotic bodies than wild-type controls. In normal mice, p27 ^Kip1 ^is evident in a subset of receptor and presynaptic taste cells beginning about 3 days post-injection, correlating with the onset of taste cell maturation. Loss of gene function, however, does not alter the proportions of distinct immunohistochemically-identified cell types.

**Conclusions:**

p27^Kip1 ^participates in taste cell replacement by regulating the number of precursor cells available for entry into taste buds. This is consistent with a role for the protein in timing cell cycle withdrawal in progenitor cells. The equivalence of mutant and wild-type taste buds with regard to cell number, cell types and general structure contrasts with the hyperplasia and tissue disruption seen in certain developing *p27^Kip1^*-null sensory organs, and may reflect a compensatory capability inherent in the regenerative taste system.

## Background

The sensory cells of mammalian chemosensory systems are unusual in that they have a limited lifespan and thus must undergo regular replacement throughout life [1-6]. In the taste system, these sensory cells are located within end organs called taste buds, which are located in 3 distinct sets of papillae in the tongue epithelium, as well as on the palate and epiglottis. Taste buds are integrative structures containing diverse cell types that interact with each other and with afferent nerve endings in ways that are complex, and as yet not fully understood [7,8]. The diversity of cells in the taste bud was first appreciated as morphological, and four cell classes, Types I through IV, were defined on the basis of cell shape, apical specialization and organelle ultrastructure [9-11]. Further studies in rodents have reported many functional and gene/protein expression differences, which has led to a functional classification of taste cells that also correlates with some distinguishing morphological features. Thus, glial-like taste cells, or Type I cells, express enzymes for inactivation and uptake of transmitters (i.e., GLAST; NTPDase2) and may participate in salty transduction [12-14]. Receptor cells, also called Type II cells, express taste receptor and intracellular signaling proteins (e.g., T1Rs and T2Rs, PLCβ2 and TrpM5) involved in sweet, bitter and umami transduction and can release ATP to potentially communicate with afferent nerves [15-22]. Type III cells are presynaptic cells, the only type to form classical synaptic contacts with afferent fibers, and evidence indicates that they transduce sour stimuli [19,23-25]. Cells immunoreactive for NCAM, serotonin and synapse-related proteins, such as the target SNARE protein SNAP-25, are primarily of this type [23,26-28]. Taste buds also contain round cells restricted to the basal areas (Type IV cells) that are thought to represent undifferentiated precursors for the mature cell types [5,29,30].

The challenge facing the adult taste system is to maintain optimal numbers of morphologically and functionally diverse cell types, and appropriate cell-cell and cell-afferent nerve interactions, so that sensory function is stable while cellular replacement is ongoing. In particular, cell proliferation must be balanced with taste cell type specification and maturation, incorporation into functional circuits, and ultimately, cell death. The mechanisms that coordinate these ongoing processes are not well understood. Among the important unknowns are the source and properties of the regenerative cells. Developmentally, taste bud cells arise from the local epithelium, rather than neuroectoderm or neural crest, and recent fate mapping studies have shown that Sonic Hedgehog (Shh)-expressing cells in mid-gestation embryos have the properties of taste cell progenitors [31-33]. However, Shh-descendent taste cells disappear early in adult life and, consequently, progenitor populations in developing and mature mice may be different [33]. As far as the adult epithelium is concerned, there is disagreement about whether long-term progenitors are located extragemmally or within taste buds. Based on immunohistochemical and lineage tracing experiments, keratin14-immunoreactive cells in the basal epithelial layer are thought to represent a bipotential progenitor cell population giving rise to both taste cells and surrounding keratinocytes, with the level of the transcription factor Sox2 believed to be critical for this cell fate decision [34-36]. On the other hand, some studies suggest that the long-term progenitor population is among the basal Type IV cells within the taste bud [30,37], and even "mature" taste cells may themselves divide and contribute new taste cells [37]. A similar lack of consensus prevails regarding cell lineages of mature taste cell types and which types undergo death and replacement [29,30,38]. However, it is generally agreed that at the end of a limited lifespan, taste cells are dismantled by apoptosis, involving activation of p53, Bax and Caspase 2 [39-42].

Although many specifics remain unknown regarding taste cell replacement, inferences based on mechanisms known to couple cell proliferation and differentiation in other vertebrate cell lineages would suggest that the proximate genes that regulate this process are likely to include those that control the cell division cycle. Progenitor cell division is driven by mitogenic extracellular signals, such as growth factors, of which many are expressed in adult taste buds [43-46]. These signals alter the expression or activity of proteins that constitute the core cell cycle machinery, including cyclin-dependent kinases (Cdks), which trigger different phases of the cell cycle [47,48], and proteins that regulate Cdk activity positively (e.g., the cyclins) or negatively (e.g., the Cdk inhibitors) [49,50]. The Cdk inhibitors, and in particular the Cip/Kip family member p27^Kip1 ^(hereafter referred to as p27), have been shown to be important in timing the onset of cell cycle withdrawal during cell differentiation [51-54]. Defects due to loss of *p27 *function, including increases in both cell number and organ size, and gain of function defects, such as reduced cell number, support the importance of this protein in arresting mitosis in various organs of the body [55-59]. Interestingly, although several Cdk inhibitors from the Ink4 and Cip/Kip families have been detected in taste epithelium *in vitro *[46], immunohistochemical studies in adult rodent taste buds have indicated that p27 is the primary member of its Cdk inhibitor family to be expressed in taste buds [60].

In the present study, we examined the effects of a *p27*-null mutation on the process of taste cell replacement in the lingual papillae of the mouse. To gain insight into the mechanisms regulating turnover of taste cells, we asked if the absence of this Cdk inhibitor alters precursor cell availability, and whether this influences the size of buds and/or the numbers of taste cells they contain. We also asked whether this mutation alters the representation of the major functional classes of taste cells. Our results indicate that p27 is an important factor regulating taste cell turnover. In its absence, the rate at which new cells appear in the taste bud, and the rate at which cells die, are substantially increased, while taste bud size and structure remain unaltered.

## Results

### Taste bud number and size in *p27*-null mice

**Figure 1:**
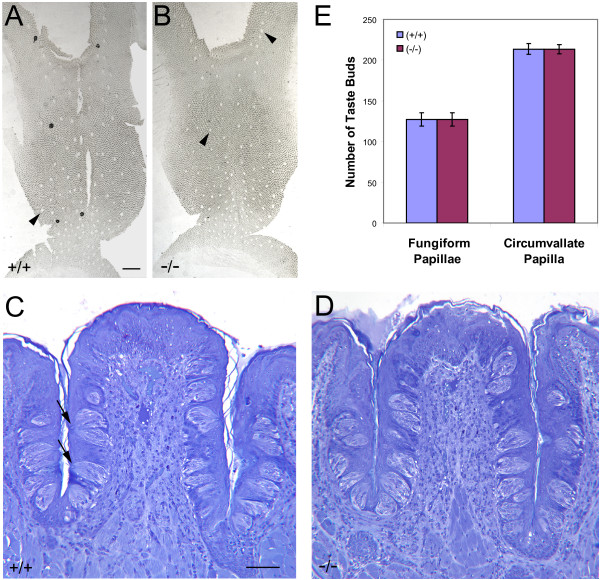
**Analysis of taste bud number and distribution in lingual papillae from 5-8 week-old wild-type and *p27*-nullizygous mice**. (A, B) Flat-mounts of superficial epithelial layers from *p27^+/+ ^*(A) and *p27*^-/- ^(B) tongues. Locations of individual FG papillae are visible as round or oval openings in the tissue (arrowheads). These were counted to estimate FG papillae number. (C, D) Light micrographs of Toluidine Blue-stained JB-4 sections showing wild-type (C) and nullizygous (D) CV papillae. The lingual epithelium and the distribution of taste buds are similar in both genotypes. Arrows in (C) point to individual taste pores, which were counted in serial sections to estimate the number of CV taste buds. (E) Total numbers of FG and CV taste buds based on counts of papillae and taste pores, respectively. Graph of means ± SEM for 4 replicates. (Student's *t*-test; *t *= 0.98 (FG) and 0.97 (CV), *df *= 6; p > 0.10) Scale bar (A and B): 100 μm. Scale bar (C and D): 50 μm.

We initially compared tongue tissues from *p27^+/+ ^*and *p27^-/- ^*mice using routine morphological techniques. The distribution and number of fungiform (FG) papillae on the dorsal anterior surface was assessed by stripping the superficial epithelial layer and mounting it flat for microscopic examination. In these preparations, the location of each papilla is evident as a gap in the tissue. As seen in Figure 1A and 1B, the arrangement and pattern of these structures is similar in tongues of both genotypes. The total number of papillae, and by inference the number of taste buds, is virtually identical in each group (Figure 1E). The number of FG papillae in these mice (127.25 ± 6.8 and 127.0 ± 5.7 in wild type and mutant, respectively) is very close to that reported for related strains of mice (e.g., 115.67 ± 7.75 [61]; 128 ± 4.3 [62]).

Analogous data were obtained for the circumvallate (CV) papillae of wild-type and *p27*-nullizygous mice. Viewed in thick plastic sections, these papillae appear very similar (Figure 1C and 1D). The overall thickness of the mutant stratified epithelium is comparable to that of the wild-type, and contains morphologically typical taste buds distributed in a single row on opposite sides of the papillary cleft. Quantitative assessment of taste pores in serial sections indicates that the number of buds per papilla is essentially the same in the two types of animal (Figure 1E). The total number of CV buds in these mice (213.8 ± 8.0 and 213.3 ± 8.3 in wild type and mutant, respectively) is also similar to previously reported mouse CV taste bud number (197, SD = 27 [63]).

**Figure 2:**
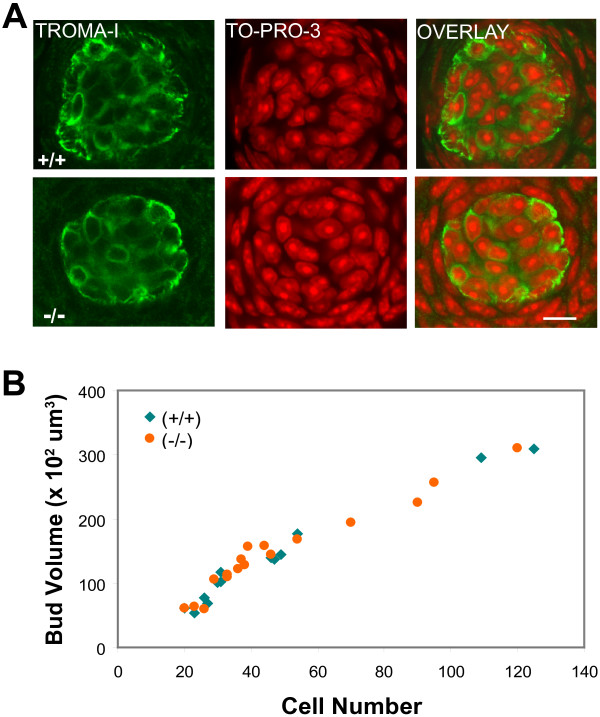
**Comparison of volume and cell number in FG taste buds from 5-6 week-old wild-type and *p27*-nullizygous mice**. (A) Confocal fluorescence images of single optical sections through a taste bud from a *p27^+/+ ^*(+/+) and a *p27*^-/- ^(-/-) anterior tongue whole-mount illustrating co-labeling with antibody to cytokeratin 8 (TROMA-I; green) and the nuclear stain TO-PRO-3 (red). The cytokeratin 8-defined perimeter was used for calculations of bud profile area, and taste bud volume was estimated by summing areas in all optical sections through a single bud. Cell nuclei lying within the bud perimeter were counted to estimate taste cell number. (B) Plot of taste cell number versus taste bud volume in papillae from both genotypes. Regression analysis indicates that the relationship between these parameters is nearly identical for the two groups (wild-type r = 0.98, mutant r = 0.97). Scale bar: 10 μm.

To determine taste bud size and the relationship between taste cell number and bud volume, whole-mount preparations of the epithelium (minus the superficial layer) from the anterior tongue were stained and viewed by confocal microscopy (Figure 2A). The single taste bud in each FG papilla was scanned in its entirety for counting taste bud cells, using immunolabeling with the TROMA-1 antibody against cytokeratin 8 to visualize bud boundaries and the nuclear stain TO-PRO-3 to identify all nuclei located inside the boundaries. The relationship between taste cell number and bud volume in FG papillae is indistinguishable in the two genotypes, indicating that average cell size is the same in both cases (Figure 2B). Furthermore, the range of taste bud volumes was the same for mutant and wild-type animals. Thus, despite a 15-20% increase in overall tongue dimensions (data not shown), the number, size and distribution of taste buds themselves are unaffected in *p27*-null mice.

### Increased taste cell turnover following *p27 *ablation

**Figure 3:**
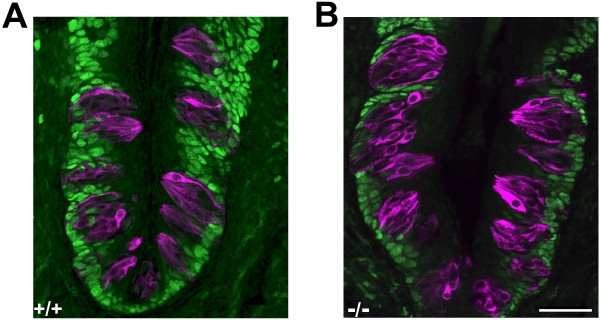
**Proliferating cells in CV papillae from 5-6 week-old wild-type and *p27*-nullizygous mice**. Confocal images illustrating anti-PCNA and TROMA-I double-labeling in wild-type (A) and mutant (B) mice. Taste cells are visualized by magenta TROMA-I staining. Anti-PCNA-labeled cycling cells (green) are found only basal or lateral to taste buds in both genotypes. Maximum projection images representing z-series of 10 optical sections separated by 1 μm steps. Scale bar: 50 μm.

Because of the established role of p27 as an anti-proliferative factor, we examined the effect of p27 loss on cell cycle progression within CV taste buds. Double-labeling with anti-Proliferating Cell Nuclear Antigen (PCNA) and TROMA-I antibody demonstrates that, as expected, in wild-type mice cycling cells are restricted to the basolateral perigemmal region of the papilla (Figure 3A). This pattern is also evident in mutant papillae (Figure 3B), indicating that the mutation does not disrupt nor displace the dividing cell population.

**Figure 4:**
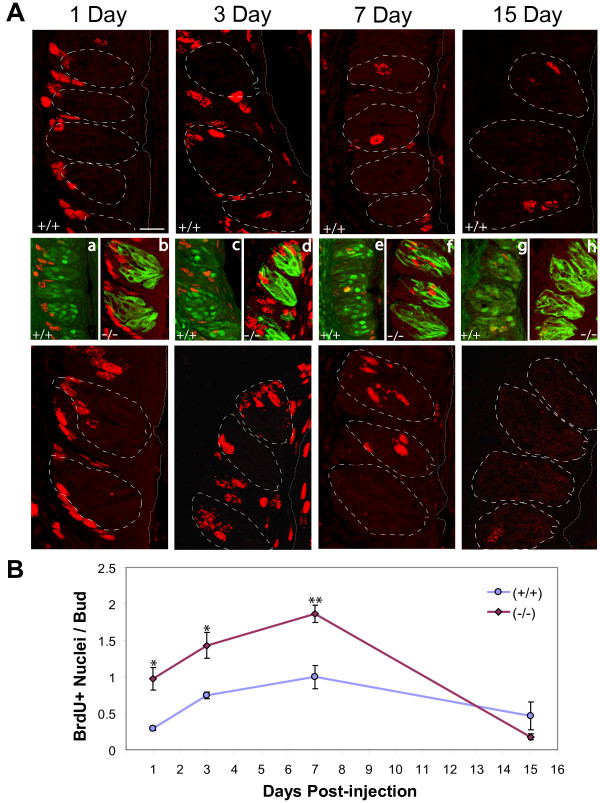
**BrdU incorporation in taste epithelium of 5-6 week-old wild-type and *p27*-nullizygous mice**. (A) Anti-BrdU immunolabeling of frozen sections 1, 3, 7 and 15 days following intraperitoneal injection of the BrdU. Labeled nuclei in wild-type (+/+) and mutant (-/-) CV papillae are evident as red ovoids. In large panels, dashed lines indicate taste bud boundaries; dotted lines demarcate the papilla surface. Maximum projection images from z-series of 10 optical sections, 1 μm steps. (a) - (h) Confocal overlay images of fluorescence double-labeling for BrdU and either p27 (in a, c, e and g) or TROMA-1 (in b, d, f, and h) (green), showing the taste buds corresponding to the dashed outlines in the wild type (+/+ panels, above) or the mutant (-/- panels, below), respectively. (B) Plot of mean number (± SEM) of BrdU-positive nuclei per bud for 3 replicates at each time point. (ANOVA: F = 9.69, p < 0.001). * indicates p < 0.02 and ** indicates p = 0.001 in post-hoc comparisons to wild-type. Scale bar: 10 μm.

To evaluate S-phase progression specifically, *p27^+/+ ^*and *p27^-/- ^*mice were injected with the synthetic thymidine analogue bromodeoxyuridine (BrdU) and their CV papillae analyzed by immunohistochemistry 1, 3, 7 or 15 days later for uptake of analogue into DNA. By our counting criterion, labeled cell nuclei are seldom observed within taste buds of wild-type animals on day 1 post-injection; however, BrdU-positive nuclei in mutant buds are significantly more frequent (Figure 4A). An almost two-fold difference in intragemmal cell labeling is seen at both days 3 and 7 (Figure 4B). By day 15, however, labeled nuclei are rare in both groups, and the difference between them has disappeared.

**Figure 5:**
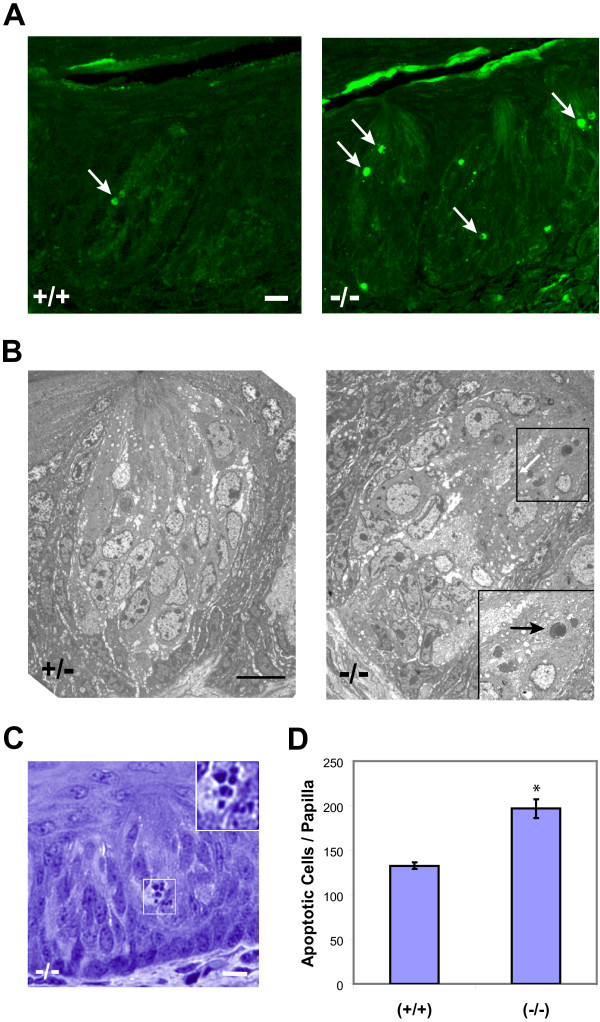
**Increased apoptosis in taste buds of *p27*^-/- ^mice**. (A) TUNEL labeling of wild-type (+/+) and *p27*-nullizygous (-/-) taste buds from CV papillae at 5 weeks of age. Papilla surface is up. TUNEL-positive nuclear fragments (arrows) occur more frequently in taste buds from mutant animals. Note the small size and condensed appearance of the labeled fragments. (B) Electron micrographs of CV taste buds from *p27*-heterozygous (+/-) and -nullizygous (-/-) mice. The distinct apoptotic body in the boxed area of the mutant taste bud is shown at higher magnification in the inset (arrow). (C) Light micrograph of a JB-4-embedded thick section from a *p27*-nullizygous CV taste bud, illustrating a dying cell with the characteristic apoptotic bodies resulting from nuclear fragmentation (boxed region). These condensed fragments, seen at higher magnification in the inset, are notably smaller and denser than the nuclei evident in the intact cells of the bud. (D) Quantitation of taste bud apoptotic bodies seen in serial JB-4 sections through CV papillae, plotted as total apoptotic cells in whole papillae (means ± SEM for 5 replicates in each genotype). The number of apoptotic cells in *p27*^-/- ^mice is approximately 1.5 times greater than in the wild-type. * indicates p = 0.005. Scale bar: 10 μm.

These results suggest that, although increased numbers of cells are initially generated in *p27-*null mutants, cell elimination processes equilibrate the numbers that survive to day 15 in the taste buds of the two genotypes. To assess this, we compared cell death in CV papillae of wild-type and mutant mice using the TUNEL assay, in which end-labeling of the large number of fragments generated during DNA degradation in apoptosis can be visualized histochemically. In wild-type taste buds, positively-labeled nuclei or nuclear fragments are occasionally observed (Figure 5A, left panel); however, in mutant papillae these structures occur more often, with at least one example per taste bud (Figure 5A, right panel). Also, in electron micrographs, more apoptotic bodies are evident in specimens from *p27^-/- ^*mice (Figure 5B). Quantitation at the light microscope level of the frequency of pyknotic cells containing apoptotic bodies within taste buds (e.g., Figure 5C) indicates that these structures are seen 50% more often in mutants, as compared to normal tissues (Figure 5D). Together, these data indicate that there is an increase in cell turnover in mutant taste buds.

### Functional marker protein expression in *p27^-/- ^*mice

**Figure 6:**
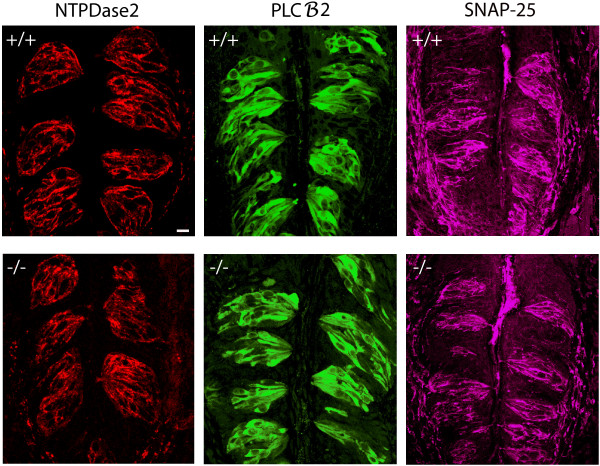
**Expression of taste cell proteins in *p27^+/+ ^*(+/+) and *p27*^-/- ^(-/-) CV taste buds**. Immunohistochemical labeling of NTPDase2 (red), PLCβ2 (green) and SNAP-25 (magenta) in taste cells indicates qualitatively similar prevalence and distributions of the three proteins and the cells expressing them in the two genotypes. Confocal maximum projections of 10 (PLCβ2 and SNAP-25) or 6 (NTPDase2) optical sections from 1 μm step z-series. Scale bar = 10 μm.

To assess whether the increased turnover rate in mutants affects the proportions of cells in each functional class, we examined expression of NTPDase2, PLCβ2 and SNAP-25 in sections of wild-type and mutant CV papillae using immunohistochemistry. The labeling patterns observed for these marker proteins are qualitatively indistinguishable between the genotypes in distribution and intensity (Figure 6). Counts of PLCβ2-labeled cells, whose borders are easily identified in these sections, produced means of 6.3 (SEM ± 0.22) and 6.2 (SEM ± 0.25) labeled cells per taste bud profile in wild-type and mutant mice, respectively.

**Figure 7:**
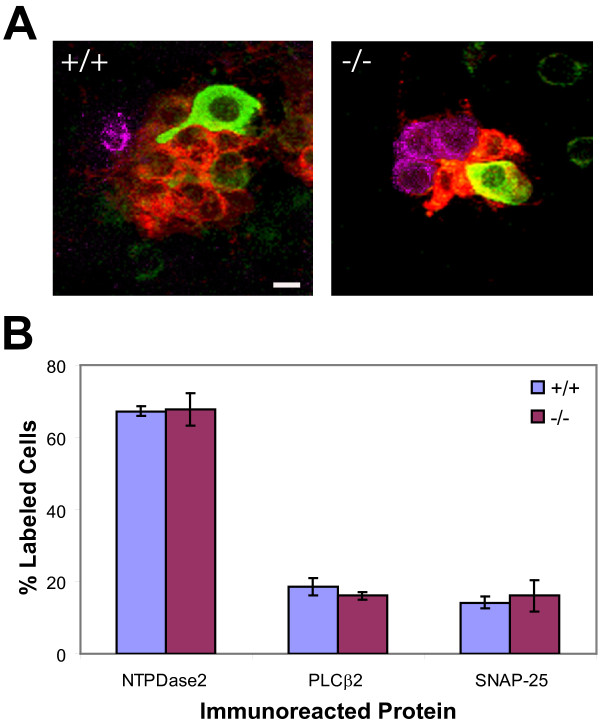
**Taste cell protein expression in dissociated preparations of wild-type and *p27*-nullizygous CV papillae**. (A) Confocal micrographs of small taste cell aggregates from wild-type (+/+) and mutant (-/-) CV papillae that were surgically isolated, enzymatically dispersed onto glass slides and processed for triple-label immunohistochemistry using antibodies to NTPDase2 (red), PLCβ2 (green) and SNAP-25 (magenta). (B) Quantitation of cell labeling for each of the specific proteins, expressed as the percentage of total labeled cells in the preparation. Chi-square test indicates that there is no significant difference between groups with respect to the relative frequency distributions of cells expressing individual proteins. No cell was labeled by more than one antibody in these preparations. (means ± SEM for 3 replicates in each condition; χ2 = 2.348; p = 0.309). Scale bar: 10 μm.

Labeling with antibodies to NTPDase2 or SNAP-25, on the other hand, did not demarcate individual cell boundaries adequately for quantitation of immunoreactive cells. To overcome this technical limitation, we developed an enzymatically dispersed CV papilla preparation in which individual epithelial cells and small clusters of these cells can be visualized and more easily distinguished from one another. Figure 7A comprises representative images of preparations that have been labeled with antibodies directed against NTPDase2, PLCβ2 and SNAP-25. In small clusters from *p27^+/+ ^*and *p27^-/- ^*mice, cells containing each of the three marker proteins are evident. Counts of cells labeled for each protein in three preparations from each genotype demonstrate that the percentage of labeled cells containing NTPDase2, PLCβ2 or SNAP-25 is virtually identical in mutant and normal papillae (Figure 7B). Thus, an appropriate representation of these different cell types is maintained in mutant taste buds.

### p27 expression in taste cells

**Figure 8:**
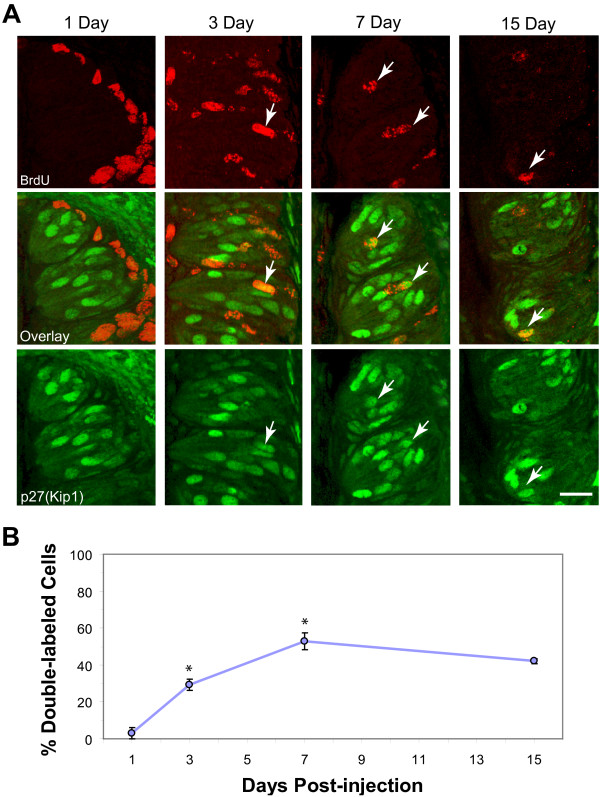
**Time course of p27 expression examined by BrdU birth-dating analysis**. (A) Confocal images (maximum projections, z-series of ten 1 μm steps) of anti-BrdU (red) and anti-p27 (green) immunolabeling in CV papillae of wild-type mice at 1, 3, 7 and 15 days following intraperitoneal injection of the BrdU. Papilla surface is to the left. No incorporation of BrdU is seen in the p27-immunoreactive taste cell population at 1 day post-injection. Co-labeled cells are evident on days 3, 7 and 15. (B) Percent (± SEM) BrdU-immunoreactive nuclei that are co-labeled for p27 with increasing cellular age. Data (means ± SEM) from 3 mice at each time point. * indicates p ≤ 0.005 compared to the previous time point. Scale bar: 20 μm.

In several neuronal systems, p27 is upregulated in development coincident with the transition from proliferating to differentiating states. To assess when in the life cycle of a taste cell p27 is expressed, we employed birth-dating analysis. Wild-type mice were injected with BrdU and their CV papillae examined 1, 3, 7 and 15 days later for cells immunoreactive for both BrdU and p27. At one day post-injection, essentially no co-labeled cells are found (Figure 8A, B). BrdU-labeled cells are restricted to the basal epithelial layer, while p27-labeled cells are observed only within the taste bud itself. However, at day 3, when a substantial number of BrdU-expressing nuclei are present in the bud, roughly a third of these nuclei also express p27. By day 7 after injection, over half of the newly-generated intragemmal cells co-label for p27. Subsequently, there is a slight decline in the percentage of BrdU-labeled cells that are p27-immunoreactive at 15 days.

**Figure 9:**
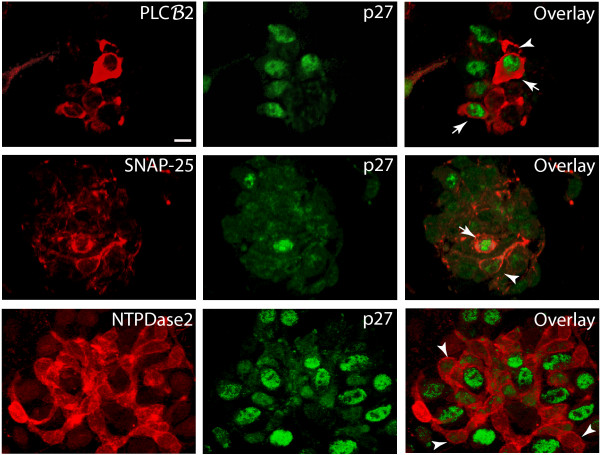
**Co-expression of p27 and taste cell proteins in dissociated CV papillae from *p27^+/+ ^*mice**. Examples of PLCβ2- and SNAP-25-immunoreactive cells that co-label for p27 are indicated by arrows, while single-labeled cell bodies are marked by arrowheads. Anti-NTPDase2-labeled profiles (red) that appear to be cell bodies are negative for nuclear p27 immunoreactivity (arrowheads), while none of the positively-labeled p27 nuclei (green) is surrounded by cytoplasm of an NTPDase2-labeled cell. Images are maximum projections of 7 optical sections taken at 1 μm intervals. Scale bar: 10 μm.

Double-labeling for p27 and each of the three marker proteins was carried out in dispersed cell preparations of wild-type CVs and evaluated using confocal microscopy (Figure 9). p27-immunoreactive nuclei were found in many PLCβ2-expressing cells, as well as in some SNAP-25-expressing cells. However, we were not able to demonstrate co-localization of p27 and NTPDase2 in these preparations.

## Discussion

In this study, we examined the consequences of *p27 *gene inactivation for maintenance of adult lingual taste buds. To date, p27 is the only member of the Cip/Kip family of Cdk inhibitors known to be produced by taste cells [60]. Given the important anti-proliferative role played by this cell cycle regulator in other sensory and neural systems, and the effects on tissue organization that result from disruption of its corresponding gene [58,64-71], we predicted that absence of p27 would have consequences for taste cell number and/or taste bud size. Our experiments, however, show that loss of a functional *p27 *gene produces little in the way of overall structural changes in the taste epithelium. This lack of an overt phenotype, nevertheless, belies substantial alterations in taste cell turnover.

One alteration we see is a greater proliferation of precursors in taste epithelium of *p27^-/- ^*mice. BrdU incorporation studies reveal a 2-fold increase in the number of S phase-labeled cells within mutant taste buds during the first week after BrdU injection. Evidence to date has generally supported the view that taste cell precursors arise from progenitors that lie outside of the taste bud and enter the bud in an immature state, as basal cells, to undergo maturation [[1,2,5,29]; however, see [37]]. Within this framework, our data indicate that, in mutants, more precursor cells are entering taste buds per unit time. This interpretation is consistent with results obtained in other systems indicating that p27 participates in regulating the timing of cell cycle exit, and, in this role, determines in part the number of cells that will go on to differentiate [51,54,72]. Clearly, p27 is not the only factor important for cell number determination, as cell cycle exit and differentiation do continue to take place in its absence. Recent *in vitro *studies of mRNA expression have identified other regulators that are present in the taste epithelium and thus are candidates for a role in contributing to, or partially substituting for p27 in, supporting these processes [46,73].

In parallel with an increase in the number of taste cell precursors incorporated in *p27^-/- ^*taste buds, we observed an elevated level of cell death in mutant buds. This was initially evident as a decline of BrdU-immunopositive cells from twice as many as in wild-type buds at day 7, to an equal number at day 15 post-injection. It was also confirmed by direct measurement of significantly greater numbers of apoptotic bodies in mutant taste buds. Thus, the fact that there are no overall changes in taste bud size and cell number with this mutation could be explained by a coincident change of equal magnitude in these two processes, which would be expected to counteract each other. Although measurements of apoptotic bodies in mutants indicate only a 33% increase in the steady state number of dying cells, compared to a 100% increase in the number of BrdU-labeled cells, direct comparison of the two measures is not necessarily warranted. Apoptotic bodies represent only those cells caught in the brief final stages of apoptosis, which are typically removed quickly from the tissue; newly generated, BrdU-labeled cells, on the other hand, persist for much longer. Our counts, therefore, very likely underestimate the amount of ongoing cell death in taste buds, as well as the relative change in this process in mutants. Together, our findings imply that removal of p27 from the system results in a state of chronically high cell flux through the taste buds.

Increased apoptosis in *p27^-/- ^*taste buds can be interpreted as a homeostatic response to an increased input of precursors into buds ("push"), due to an enhanced production of cells by taste progenitors. In this view, as long as the cell death machinery is intact, taste buds are capable of autonomously regulating their cell numbers, independently of progenitor proliferation rate. However, loss of p27 could additionally or alternatively be influencing cell death directly. In differentiating mouse embryonic stem cells *in vitro*, deletion of p27 increases the number of cells that undergo apoptosis, indicating that this Cdk inhibitor normally has a survival function in these cells [74]. If p27 plays a similar role in the taste system, a null mutation could promote cell death and induce the requirement for enhanced generation of replacement cells to maintain the taste bud ("pull"). In either case, our results suggest that taste buds may be subject to growth compensation, and that there are global mechanisms at work to regulate the size of taste buds within strict parameters [75,76].

Our experiments combining BrdU birth-dating with immunolabeling for p27 define more precisely the association of p27 up-regulation with the initiation of phenotypic maturation in taste cells. In the newly generated population, p27 is expressed strongly after about three days post-injection. Others have reported that the period between 2 and 5 days post-injection sees the onset of expression of various proteins associated with phenotypic and functional maturity, including the signaling molecules gustducin [77-79] and PLCβ2 [80,81], the synapse-related SNAP-25 [81], cytokeratins 8 and 18 [35,82], and T1R3 [78]. Peak expression of these molecules has been reported to occur at about 6-7 days post-injection [77,80]; in our experiments, the percentage of BrdU-labeled cells that were immunoreactive for p27 continued to increase to day 7.

Interestingly, however, while high levels of p27 expression are temporally associated with cell maturation, the protein is present in less than 50% of BrdU-labeled cells at any time. Theoretically, this limited expression could reflect restriction to a particular stage of cell maturity, or to a discrete cell type (or both). In the present studies, we observed p27 co-expressed along with either PLCβ2 or SNAP-25, proteins which are mostly present in receptor and presynaptic cells, respectively [16,19,26]. On the other hand, we could not verify co-labeling for p27 in NTPDase2-positive cells, which are likely to be Type I cells [13]. The simplest interpretation of these data is that p27 expression is restricted to and maintained in maturing and mature cells in the bud, excluding the glia-like supporting cell population. Curiously, this is distinctly different from the pattern observed in the peripheral visual and auditory systems. Here, although p27 is expressed in nearly all cells at the time of differentiation, including sensory cell precursors, maintained expression of p27 in adults is limited to the mature supporting/glial populations [58,65,66,68,83,84].

The reason for sustained, high expression of p27 in certain groups of taste cells and not others is unclear. There is evidence in developing neuronal systems that different Cdk inhibitors may time cell cycle exit in distinct subsets of progenitor cells that give rise to different cell types [58,85]. This raises the possibility that mature non-p27-expressing taste cells arise from a progenitor population in which a different Cdk inhibitor performs this function. The Cip/Kip family member p21 is also expressed in mouse taste buds, but p21-labeled cells represent only a small fraction of taste cells [60], and thus seem unlikely to correspond to the large population of non-p27-expressing cells we see in the bud. A possible role for other Cdk inhibitors (i.e., the INK4 family) in this regard requires further investigation. Also, in non-gustatory sensory systems, the cells that continue to express p27 in adulthood generally retain the ability to re-enter the cell cycle and proliferate in response to injury or other pathological conditions, a process which includes down-regulation of p27 [67,86]. While mature taste cells are generally considered to be post-mitotic, evidence for a small fraction of cycling cells within buds has recently been reported [37], suggesting that in some circumstances certain taste cells may retain this ability. Thus, it is possible that one role of this Cdk inhibitor is to generally restrict cell cycle reentry in functionally mature cells. However, we found little evidence for ectopic cell division within taste buds of *p27*-null animals, again suggesting that if p27 plays such a role, it is not acting alone.

While p27 is clearly important in taste bud turnover, our data indicate that it does not have a direct role in determining taste cell fate. Triple-label immunohistochemical studies show that lack of p27 does not alter the proportion of cells that label for each of three functional cell type marker proteins. This finding is in agreement with the general consensus in developing systems that the role of p27 is restricted to regulating cell number and not cell differentiation [58,65,85]. In our studies, the dispersed CV papilla preparation proved a reliable method for phenotypic evaluation of taste cells by immunolabeling. In quantifying the differential labeling, we found that the variability was low within groups as well as between the two groups of animals, and that the proportions of differentially-labeled cell types we found were broadly within the ranges that would be expected, based on data from intact mouse CV [87]. Importantly, we did not find any double-labeled cells in these preparations, indicating that the antibodies we used labeled distinct taste cell populations.

Overall, these studies demonstrate, for the first time, that the developmentally important cell number regulator p27 also plays a role in the regenerative cell system of adult mouse taste buds. Given its demonstrated importance in several developing sensory systems, it seems likely that p27 might function in the developing taste epithelium as well. However, lack of the protein from birth does not ultimately alter the number or organization of taste papillae, buds or cells. Nonetheless, because in adults the protein seems largely to regulate the dynamics of taste cell life cycles, a similar effect may be evident as alterations in developmental timing and, consequently, sequencing of events leading to a functional peripheral taste system. Defining the role of p27 in the development of this system is the object of our continuing studies. Finally, the implications of chronic increase in cell flux through the taste bud for gustatory function, and for maintenance of the taste epithelium in aging animals, are unclear. Resolution of these questions also awaits future study.

## Conclusions

Our data demonstrate that p27 plays an important role in the dynamics of taste cell turnover. Germline loss of *p27 *leads to enhanced generation and entry into taste buds of undifferentiated cells, consistent with a function for the protein in timing cell cycle withdrawal in taste cell progenitors. However, this increase in the availability of potential taste cells is offset by an enhanced rate of cell death. It remains undetermined whether excess cells that enter the bud all go on to differentiate, or if they are eliminated as immature cells. The overall result is no change in taste bud size or cell number. Thus, regulatory mechanisms presumably exist at the level of the tissue to ensure that bud dimensions are ultimately constrained.

In contrast to its involvement in regulating cell cycle exit, p27 does not appear to play a role in taste cell differentiation. Up-regulation of the protein corresponds temporally with taste cell maturation, and it is only expressed in subsets of Type II and Type III cells, and apparently not at all in Type I cells. Despite this differential expression, loss of gene function does not perturb the relative proportions of cell phenotypes.

## Methods

### Mice and genotyping

Hemizygous *p27 *mice (+/-), on a C57BL/6 background (B6.129S4-*Cdkn1b^tm1Mlf^*/J; [55]), were purchased from The Jackson Laboratory (Bar Harbor, ME) and used to set up a breeding colony. Animals were housed in the Division of Laboratory Animal Resources at ETSU College of Medicine in accordance with Association of Assessment and Accreditation of Laboratory Animal Care (AAALAC) guidelines. Tail snips were taken from all mice on or before 4 weeks of age and genomic DNA extracted and purified using an Extract-N-Amp Tissue PCR Kit (Sigma-Aldrich, St. Louis, MO). Genotypes were determined by performing PCR amplification of the wild-type and mutant *p27 *alleles [55]. One common sense primer (5'-TGG AAC CCT GTG CCA TCT CTA T) was used to detect both alleles, while the antisense primers employed were specific for the wild-type allele (5'-GAG CAG ACG CCC AAG AAG C) and the *neo*-disrupted allele (5'-CCT TCT ATG GCC TTC TTG ACG). PCR was performed for 40 cycles with an annealing temperature of 55°C [88]. Oligonucleotide products were then resolved on a 2% agarose gel and visualized after staining with Sybr Gold (Invitrogen, Carlsbad, CA). The wild-type allele resulted in amplification of a 1300-bp product and the null allele a 600-bp product [55].

### Histological analysis of circumvallate papillae, taste bud quantitation and electron microscopy

Mice (5-6 weeks old) were euthanized by CO_2 _asphyxiation and tongue tissues surgically removed and immersion-fixed overnight in 4% glutaraldehyde in 0.1 M phosphate buffered saline, pH 7.4 (PBS). Circumvallate papillae and immediately surrounding tissues were dissected from the tongues and rinsed several times in buffer in preparation for plastic embedding. For routine histology, tissues were dehydrated in graded ethanols prior to infiltration in JB-4 embedding medium (EM Sciences, Hatfield, PA). Polymerization was carried out according to the manufacturer's instructions and the tissue blocks were stored under dessicant until sectioned. Serial sections (approximately 3 μm) were obtained in a plane perpendicular to the tongue surface, and stained with 2% Toluidine Blue O (Sigma-Aldrich). For documentation, digital images of the stained sections were acquired using a Spot Insight 4 camera system (Diagnostic Instruments, Inc, Sterling Heights, MI) mounted on a Leitz Laborlux S microscope with a 20X objective, and processed using Photoshop CS2 (Adobe Systems, San Jose, CA).

Morphometric analysis was performed using an Olympus BH-2 microscope equipped with a 40X objective. The number of taste buds in each CV papilla was determined by counting taste pores in serial sections through the papilla. Taste pores appearing for the first time in each section were counted, with great care taken to ensure that the same pore was not counted in more than one section. Typically, one taste pore appeared in 2 or 3 consecutive sections; no taste buds with more than one taste pore were observed in CV papillae. Taste bud counts were performed on 5 mutant and 5 wild-type papillae from male and female mice, and values were averaged within the groups for between-group statistical comparison.

For transmission electron microscopy, a separate group of glutaraldehyde-fixed CV papillae was post-fixed in 2% osmium tetroxide in phosphate buffer. After extensive rinsing in buffer and distilled water, specimens were dehydrated and embedded in Epox 812-Araldite (Ernest F. Fullam, Latham, NY). Thin (90 nm) sections were stained with uranyl acetate and lead citrate and examined in a Philips Tecnai 10 electron microscope (FEI, Hillsboro, OR). Images were acquired using a Mega-Plus Model ES 4.0 CCD camera (Princeton Instruments, Trenton, NJ) and processed using Photoshop.

### Counting and volume measurement of fungiform taste buds

#### Preparation of tongue epithelium flat-mounts and counting of papillae

Mice (6-8 weeks old) were euthanized and tongue tissues removed as above. Excised tongues were fixed for 1 hr in 4% paraformaldehyde in PBS. CV papillae and surrounding tissues were removed for use in other studies, and the muscle underlying the anterior tongue was trimmed as close as possible to the upper tongue surface and discarded. The remaining lingual tissue was re-immersed in 4% paraformaldehyde overnight at room temperature. It was then rinsed several times in PBS and again refrigerated overnight.

To enzymatically isolate the superficial epithelial layers, the trimmed anterior tongue was placed in 5 ml of a solution of 2% Dispase II (neutral protease; Roche, Indianapolis, IN) in PBS and agitated on a shaker for 2-3 hrs at room temperature. After an overnight rinse, specimens were placed in boiling 2% citrate buffer (pH 6.0) for 30 min, cooled to room temperature, and rinsed in PBS. The already separating superficial layer was carefully peeled away from the tongue surface under a dissecting microscope. Two slits were made laterally at the anterior edge to allow the otherwise intact sheet of tissue to be mounted flat on a slide and then coverslipped in Permount. All profiles of superficial epidermal structures corresponding to the location of FG papillae were then visualized and counted (Figure 1A &1B). Four *p27^-/- ^*and four *p27^+/+ ^*tongues from equal numbers of males and females were examined. Total counts were averaged within groups for statistical comparison between mutants and wild-type controls.

#### Immunohistochemistry in flat mounts

The tongue tissue remaining after the superficial layers had been stripped contained the intact FG taste buds, which were visualized by immunohistochemical labeling for cytokeratin 8 with TROMA-I antibody [89,90]. The prior heat treatment in citrate buffer to which the intact tongue had been subjected served as an initial antigen retrieval step in the processing protocol. Washed tissue was placed in 0.4% Triton X-100 in PBS for 18-36 hrs at 4°C, then in TROMA-I concentrate (Developmental Studies Hybridoma Bank, Iowa City, IA) diluted 1:50 in standard incubation buffer (0.4% Triton X-100 + 1% bovine serum albumin in PBS), which was the diluent in all subsequent non-wash steps, for a further 18-36 hrs at 4°C. This was followed, after PBS washes, by 2 hrs incubation in biotinylated goat anti-rat IgG (1:200; Jackson ImmunoResearch, West Grove, PA) and finally by streptavidin-conjugated DTAF (Jackson ImmunoResearch) at the same dilution for 2 hr. The tissue was then immersed in To-Pro-3 (Invitrogen), a fluorescent nuclear label, at a 1:500 dilution in PBS for 30 min at room temperature. After rinsing, the tissue was mounted in VectaShield fluorescence mounting medium (Vector Labs, Burlingame, CA) and a cover glass was carefully applied to avoid compressing the tissue, using glass shims around the edges to match cover glass height to tissue thickness. The cover glass edges were sealed with clear nail polish.

#### Confocal microscopy and data analysis

Specimens prepared for immunofluorescence microscopy were viewed in a Leica SP confocal laser scanning microscope (Leica, Heidelberg, Germany) equipped with an inverted fluorescence microscope and a 20X infinity-corrected objective. Individual taste buds were located and scanned from surface (i.e., taste pore) to base in a z-axis series of optical sections taken at 1.0 μm intervals. Taste buds were selected at random, but care was taken to sample from all regions of each tongue. Between 5 and 10 buds from each of five *p27^-/- ^*and four *p27^+/+ ^*tongues were scanned. In all cases, wild-type and mutant specimens were processed in the same manner and images were acquired using identical instrument parameters, although the total number of optical sections varied depending on the size of the bud. In the course of sampling these papillae, we encountered none containing more than one taste bud.

Only taste buds that had distinct boundaries, were fully intact and were undistorted by compression were used in the analysis. The boundary created by the TROMA-I immunofluorescence was used to define the circumference of the taste bud in each optical section through a bud. The area of each defined region was estimated using the Nucleator probe component of Stereo Investigator software (MBF Bioscience, Williston, Vermont). With this probe, each profile is modeled as a circle. Operationally, a set of 6 rays emanating from a central point were superimposed on the taste bud image and the point of intercept of each ray with the bud boundary was defined. Based on the length of each of the 6 lines from the central point to the intercept, an average radius was calculated, and an estimated area determined (A = πr^2^). Area estimates from all optical sections in the series were summed, and a volume estimate in μm^3 ^was then calculated by multiplying by the optical section thickness (i.e., 1.0 μm). A validation study was performed by measuring the diameter of the largest profile (determined from Nucleator area estimates) in the z-series of optical sections for each bud. Calculations from these measurements yielded a mean diameter of 37.0 (SD = 9.1) μm for the population, which agrees well with the previously reported mean of 42 (SD = 6) μm, as measured from 8 μm thick, brightfield sections [63]. To determine the number of cells in each taste bud, To-Pro-3 labeled nuclei contained within the defined circumference of the bud were counted. Nuclei appearing for the first time were counted in each sequential serial optical section through the bud, care being taken not to count the same nucleus in more than one section.

### Cell proliferation and cell death analyses

#### BrdU Incorporation

In order to assess the impact of *p27 *deletion on cell division, DNA synthesis was assessed by immunohistochemical detection of BrdU incorporation. *p27^+/+ ^*and *p27^-/- ^*mice were labeled by intraperitoneal injection of BrdU (50 μg/g body weight; Sigma-Aldrich) in PBS. Animals were injected twice on day 0, at 10:00 AM and 4:00 PM, and sacrificed 1, 3, 7 or 15 days later. Tongues were excised, fixed in 4% paraformaldehyde for 1 hr, and CV papillae removed and processed by published methods [60]. Briefly, tissue blocks were further fixed overnight at room temperature, washed overnight in PBS at 4°C, cryoprotected in 10% sucrose (12 hrs at 4°C) and then 20% sucrose (18 hrs at 4°C). After embedding in OCT (Tissue Tek, Torrance, CA), tissue was frozen in melting Freon 22 and stored at -70°C. Sections (6 μm) were cut on a cryostat microtome at -20°C, collected on subbed slides, dried at 37°C for 15 min and stored at -20°C for up to several weeks before further processing.

Slides removed from the freezer were dried at 37°C for 15-30 min. Prior to antibody labeling, sections were incubated in 1% Triton X-100 in PBS for 10-20 min, then washed in PBS and treated for 30 min with 1N HCl at room temperature. Specimens were neutralized immediately with 0.1 M borate buffer, pH 8.5 and finally rinsed in PBS. After treatment with 10 mM citrate buffer solution (pH 6.0) for 15 min at 98°C for antigen retrieval, slides were cooled and rinsed. To label incorporated nuclear BrdU, sections were blocked for 1 h in 10% normal serum and treated overnight at 4°C with polyclonal sheep anti-BrdU (1:500 dilution; Abcam, Inc., Cambridge, MA). Bound antibody was detected with donkey anti-sheep IgG conjugated to Alexa Fluor 555 (1:400 dilution, 1 h at room temperature; Invitrogen). To identify approximate taste bud boundaries, sections were also labeled with TROMA-I (1:100, overnight at 4°C), which was visualized with biotinylated anti-rat IgG (1:250, 2 hr) followed by streptavidin-conjugated Alexa Fluor 488 (1:600, 2 hr). Tissue immunofluorescence was viewed by confocal laser scanning microscopy.

For quantitation of BrdU labeling in taste buds, z-series of optical sections through CV papillae were collected as described above, and used to construct an extended focus image from all optical sections in the data set (i.e., a maximum projection). Typically, 35-45 serial sections were obtained from any one papilla, and every third section was scanned in the confocal microscope. Outlines of taste buds were evaluated on the confocal images, and only those extending from the base of the epithelium to its surface, and thus representing the approximate center of the bud, were selected for study. For each such bud profile, the number of BrdU-positive nuclei contained within it was recorded. Only labeled nuclei lying more than one nuclear width inside the apparent lateral or basal boundaries of the bud profile were counted as being within the bud. Data were collected from 3 mutant and 3 wild-type papillae at each of the post-injection time points, and expressed as the average number of BrdU-labeled nuclei per taste bud. Statistical analysis consisted of ANOVA (SYSTAT, SPSS, Chicago, IL) followed by Fisher's LSD multiple range test of significance between selected means.

#### Morphometry and TUNEL assays

Cell death was assessed by both morphometric and TUNEL assays for apoptotic nuclei. The serial JB-4-embedded sections used for counting CV taste buds were also used to estimate numbers of dying cells in the two genotypes. Apoptotic bodies were counted and expressed as the number per taste bud. Condensed spherical and/or crescent shaped bodies with darkly staining chromatin, with or without a surrounding cell cytoplasm and membrane, and collections of condensed, dark fragments (counted as one apoptotic body) [91,92], were included as apoptotic bodies. Care was taken to track taste buds through adjacent sections and count each bud and dying cell only once. Data were averaged for each of the two groups for statistical comparison using Student's t test.

For molecular detection of apoptotic nuclei, TdT-mediated dUTP-biotin nick end labeling (TUNEL) assays, in which DNA strand breaks are detected *in situ *via incorporation of fluorescein-dUTP by exogenously-applied terminal deoxynucleotidyl transferase [93], were performed. This method relies on the availability of large numbers of free ends of DNA fragments which can be labeled to visualize the nuclei (or their remnants) that have undergone DNA degradation during apoptosis. To maintain tissue morphology and achieve adequate staining, standard TUNEL processing was modified by incorporating a 24 hr incubation of the sections in 1% Triton X-100 in PBS, followed by 30 min in 1N HCl at 37°C and then antigen retrieval (10 mM citrate buffer solution, 15 min, 97°C). Subsequent processing followed instructions provided with the TUNEL kit (Roche Applied Science, Indianapolis, IN). Qualitative evaluation of relative levels of DNA labeling was carried out in the confocal microscope.

### Double-label immunohistochemistry

#### TROMA-I and PCNA

To identify cycling cells in both G_1 _and S phases, proliferating cell nuclear antigen (PCNA) was localized by immunohistochemistry [94,95]. Sections were processed for antigen retrieval (10 mM citrate buffer solution, 9 min, 100°C) and incubated in anti-PCNA (1:500; DAKO, Carpinteria, CA) overnight at 4°C, followed by FITC- or DTAF-conjugated anti-mouse IgG at room temperature for 2 hrs. Subsequently, tissue was reacted with the TROMA-I antibody, using the same procedure as for double-labeling with this antibody in BrdU-labeled tissue, described above. Labeled papilla sections were examined in the confocal microscope.

#### p27 co-expression experiments

The expression of p27 in functional types of taste cells within taste buds was evaluated in a series of double-label immunohistochemistry experiments. Tissue blocks containing CV papillae were excised from *p27^+/+ ^*mice, fixed in 4% paraformaldehyde and processed for frozen sectioning as described above. In most cases, cryosections (6 or 10 μm) on slides first underwent antigen retrieval (10 mM citrate buffer, pH 6.0, 15 min, 98°C) and then incubation in 10% normal serum in standard incubation buffer, followed by overnight incubation at 4°C with mouse monoclonal anti-p27 (1:100; Transduction Laboratories, Lexington, KY). Primary antibody labeling was detected by sequential application of biotinylated secondary antibody (1:400) at room temperature for 2 hrs and streptavidin-conjugated Alexa Fluor 488 or DTAF (1:600) for 1 hr. After rinsing, sections were again blocked in normal serum and incubated in primary antibody specific for either PLCβ2 (1:1000; Santa Cruz Biotechnology, Inc., Santa Cruz, CA), or SNAP-25 (1:500; Chemicon, Temecula, CA) and labeling was visualized with an IgG directed against the appropriate species conjugated to Cy3 (1:300) or Alexa Fluor 555 (1:400). Because NTPDase2 immunoreactivity was destroyed by the antigen retrieval process, sections were processed for NTPDase2 labeling prior to heat treatment. Sections were incubated in the diluted primary antibody (1:1000; Dr. J. Sevigny, CHUQ, Quebec City, Quebec, Canada) overnight at 4°C and labeling was visualized with Alexa Fluor 555-conjugated IgG (1:400). The tissue subsequently underwent antigen retrieval and immunolabeling for p27. Tissue sections were evaluated for co-labeling of individual marker proteins with p27 using the confocal microscope.

#### p27 and BrdU double-labeling

Double-labeling immunohistochemistry for BrdU and p27 was only successful when BrdU labeling was carried out first. Upon completion of the BrdU labeling procedure as described above, sections from CV papillae were blocked in normal serum and then incubated in anti-p27 antibody. Labeling was visualized using a biotinylated secondary antibody and streptavidin-conjugated Alexa Fluor 488, as above. Z-series of optical sections through the papillae were collected in the confocal microscope; typically, every third section through the papillae was scanned. Only taste bud profiles extending from the base of the epithelium to its surface were evaluated. For each such bud profile, the number of BrdU-positive nuclei, the number of cells labeled for p27 expression and the number of double-labeled cells within each bud were recorded. Data were collected from 3 or 4 wild-type specimens at each post-BrdU injection time point. The mean number of single- and double-labeled nuclei per taste bud was calculated and statistical significance was evaluated using ANOVA and post-hoc Bonferroni tests of means (SYSTAT).

### Immunohistochemistry in dissociated cell preparations from CV papillae

#### Preparation of cell dispersions

Whole tongues were excised from mutant and wild-type mice and injected with 1% Dispase II dissolved in antibiotic treated Balanced Salt Solution (BSS) [96]. Injections were made parallel to and just beneath the epidermis with the needle being inserted from the posterior (cut) end. The needle was carefully advanced to the anterior end of the tissue, avoiding contact with the CV papilla, and slowly retracted posteriorly as Dispase II was gradually released from the syringe. Following incubation for 15 minutes at room temperature in BSS, the epidermal layer was stripped from the tongue. This layer included the CV papilla. Extraneous tissue was carefully trimmed away from the two invaginations visible on the superior surface and from the two sack-like structures visible on the inferior surface. These sacks, or "clefts", were separated from each other by cutting down the midline, and then washed briefly (2 times, 5-10 sec each) in isolation buffer solution (IBS: 0.5 mM EGTA, 0.4 μM EDTA, 1% polyvinylpyrrolidone, 0.15 M NaCl, 15 mM HEPES buffer; [97]). Tissue was transferred into a microcentrifuge tube containing 100 μL of 0.05% trypsin (Calbiochem, San Diego, CA) in IBS pre-heated to 37°C, and incubated at 37°C for 10 minutes. Trypsin was then inactivated with 20 μL of 1% trypsin inhibitor (Sigma-Aldrich) in IBS. Clefts were triturated using a small mouth transfer pipette to dissociate the tissue into single cells and small cell clusters.

Suspensions of dispersed cells were centrifuged onto Cell-Tak (BD Biosciences, Bedford, MA) coated slides using a Shandon-Elliot Cytospin (SCA-0030, Shandon Southern Instruments, Surrey, England) at 2000 rpm for 3 minutes. Coated slides were prepared the day of cell dispersion following the manufacturer's protocol (i.e., 11.8 μL of Cell-Tak at 2.1 mg/mL in 5% acetic acid applied to the slide and allowed to evaporate). After centrifugation, slides were rinsed with distilled water and dried at room temperature. Slide-mounted cells were then fixed for 10 minutes in 4% PFA, washed twice for 10 minutes in PBS and stored at -20°C until used for immunohistochemistry.

#### Cell dispersion triple-labeling immunohistochemistry

Prepared slides removed from the freezer were dried at 37°C for 20 minutes, washed in PBS and blocked in 10% normal serum, followed by overnight incubation at 4°C with a combination of primary antibodies for PLCβ2 (1:1000; Santa Cruz Biotechnology, Inc., Santa Cruz, CA) and SNAP-25 (1:500; Covance, Emeryville, CA). Buffers and washes were as described above. Labeling for PLCβ2 was visualized using a biotinylated secondary antibody (1:400, 21°C, 2 hrs) and streptavidin-conjugated Alexa Fluor 488 (1:600, 21°C, 1 hr). Labeling for SNAP-25 was visualized using a secondary antibody conjugated to Alexa Fluor 647. Sections were subsequently processed for NTPDase2 (1:1000, 21°C, 2 hrs), which was visualized with an Alexa Fluor 555-conjugated IgG.

Z-axis series of optical sections were obtained on the confocal microscope at 6 locations in each dispersed cell preparation, and maximum projections of each were constructed. Locations were selected using systematic random sampling, such that the first sampled site was randomly selected, and the subsequent 5 sites determined by shifting two complete fields of view laterally and one field down, returning to the opposite or top borders when edges were encountered. All fluorescently-labeled cells were counted at each site, as well as the number labeled by each of the 3 primary antibodies, in 3 preparations from each genotype. Data for each antibody was expressed as the percentage of total fluorescent cells. Results for mutant and wild-type papillae were compared using a Chi-square test for statistical significance (MATLAB, MathWorks, Inc., Natick, MA).

#### Cell dispersion p27 co-expression immunohistochemistry

Slides with dispersed papilla preparations from wild-type mice first underwent antigen retrieval (10 mM citrate buffer, pH 6.0, 15 min, 98°C) and block in 10% normal serum, then were incubated overnight at 4°C in mouse monoclonal anti-p27 (1:100; Transduction Laboratories, Lexington, KY). Biotinylated secondary antibody (1:400; 21°C, 2 hrs) and streptavidin-conjugated Alexa Fluor 488 (1:600, 21°C, 2 hrs) were used for visualization. Sections were subsequently processed in either PLCβ2 (1:1000), NTPDase2 (1:1000), or SNAP-25 (1:500) in buffer solution and labeling was visualized with an IgG against the appropriate species, conjugated to Alexa Fluor 555 (1:400). Labeling was evaluated qualitatively using confocal microscopy.

## Authors' contributions

This study was conceived and designed by TAH and DMD, who also supervised data analysis and drafted the manuscript. LBSA and PDM developed methodologies for whole mount histology and immunohistochemistry experiments, and dispersed papillae preparation immunohistochemistry experiments, respectively. They also contributed to design and data analysis for these experiments. LBSA, PDM, MKP and JDS all participated in tissue preparation and immunohistochemistry experiments. All authors approved the final manuscript.
